# Genome-wide genetic aberrations of thymoma using cDNA microarray based comparative genomic hybridization

**DOI:** 10.1186/1471-2164-8-305

**Published:** 2007-09-03

**Authors:** Gui Youn Lee, Woo Ick Yang, Hei Cheul Jeung, Sang Chul Kim, Min Young Seo, Chan Hee Park, Hyun Cheol Chung, Sun Young Rha

**Affiliations:** 1 Cancer Metastasis Research Center, National Biochip Research Center, Yonsei University College of Medicine, Seoul, Korea; 2 Brain Korea 21 Project for Medical Science, Yonsei University College of Medicine, Seoul, Korea; 3 Department of Pathology, Yonsei University College of Medicine, Seoul, Korea; 4 Yonsei Cancer Center, Yonsei Cancer Research Institute, Yonsei University College of Medicine, Seoul, Korea; 5 Department of Internal Medicine, Yonsei University College of Medicine, Seoul, Korea

## Abstract

**Background:**

Thymoma is a heterogeneous group of tumors in biology and clinical behavior. Even though thymoma is divided into five subgroups following the World Health Organization classification, the nature of the disease is mixed within the subgroups.

**Results:**

We investigated the molecular characteristics of genetic changes variation of thymoma using cDNA microarray based-comparative genomic hybridization (CGH) with a 17 K cDNA microarray in an indirect, sex-matched design. Genomic DNA from the paraffin embedded 39 thymoma tissues (A 6, AB 11, B1 7, B2 7, B3 8) labeled with Cy-3 was co-hybridized with the reference placenta gDNA labeled with Cy-5. Using the CAMVS software, we investigated the deletions on chromosomes 1, 2, 3, 4, 5, 6, 8, 12, 13 and 18 throughout the thymoma. Then, we evaluated the genetic variations of thymoma based on the subgroups and the clinical behavior. First, the 36 significant genes differentiating five subgroups were selected by Significance Analysis of Microarray. Based on these genes, type AB was suggested to be heterogeneous at the molecular level as well as histologically. Next, we observed that the thymoma was divided into A, B (1, 2) and B3 subgroups with 33 significant genes. In addition, we selected 70 genes differentiating types A and B3, which differ largely in clinical behaviors. Finally, the 11 heterogeneous AB subtypes were able to correctly assign into A and B (1, 2) types based on their genetic characteristics.

**Conclusion:**

In our study, we observed the genome-wide chromosomal aberrations of thymoma and identified significant gene sets with genetic variations related to thymoma subgroups, which might provide useful information for thymoma pathobiology.

## Background

Thymoma is a thymic epithelial cell tumor having organotypic features and no overt cytologic atypia. Although controversy still remains, the classical histological classifications of thymoma based on the proportion of reactive lymphocytes and tumor epithelial cells have been replaced by a histogenetic classification that basically subdivides thymoma into medullary, cortical, and mixed types, according to cytological features of epithelial cells [1-5]. Several studies supported the validity of the histogenetic classification and the World Health Organization (WHO) classification adopted five subtypes of thymoma stratified in histogenetic classification [6-9]. The histological subtypes in the WHO classification have been reported to have independent prognostic significance, and types A and AB demonstrate indolent biological behavior compared with type B [6-8]. Several studies have tried to demonstrate the underlying pathogenetic mechanisms to explain the different biological behaviors of thymoma, according to the histological subtype and stages by applying several markers, but no conclusive results have been demonstrated yet [10-18].

A recent report on genetic alterations of thymoma using comparative genomic hybridization (CGH) and fluorescent in situ hybridization methods demonstrated that type B3 thymoma frequently occurred with losses of chromosomes 6, 13 q, 16 q and gains of chromosome 1 q, while type A thymoma rarely showed cytogenetic abnormalities [19]. Subsequent studies by the same group based on loss of heterozygosity (LOH) analyses inferred two different genetic pathways of tumorigenesis of thymoma, and heterogeneous genetic alterations in subtypes of thymoma, excluding type A, were identified by CGH and LOH analyses [20,21]. However, a recent CGH study identified several new chromosomal imbalances even in a significant proportion of type A thymomas [22].

The application of DNA microarray technology has provided us a high-throughput evaluation of the whole genome as well as significant genetic information at a single gene level and has enabled us to classify different neoplasms based on the characteristic genetic patterns. So far there has been no report focusing on differences of genetic alterations between subtypes of thymoma using a cDNA microarray based-CGH method (microarray-CGH). In our study, genetic alterations of all WHO-defined subtypes of thymoma were investigated using high resolution microarray-CGH followed by hierarchical cluster analyses of the data to identify specific patterns of genetic aberrations and genes associated with each subtype.

## Results

### Chromosome analysis of thymoma

**Figure 1:**
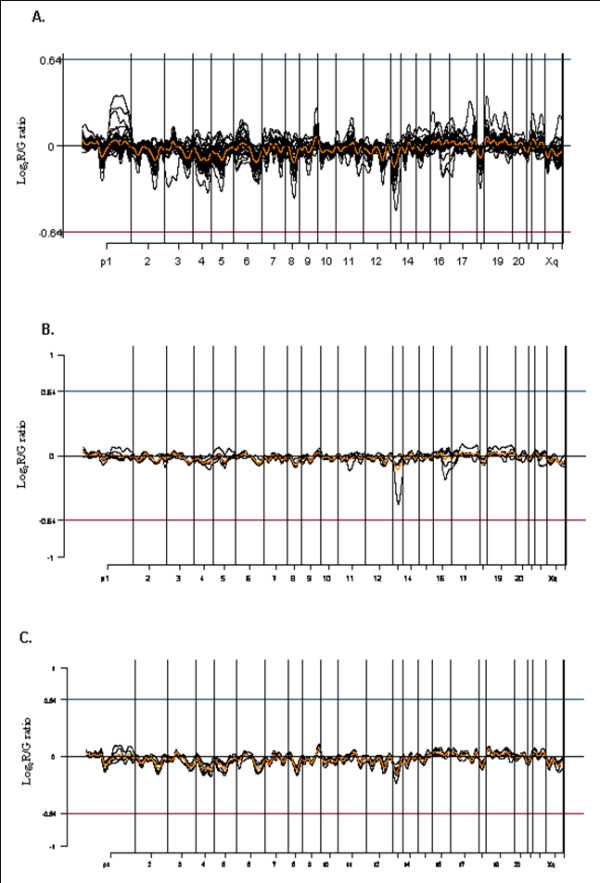
Microarray-CGH profiles using CAMVS (Chromosome analyzer and Map Viewer using S plus). Each black line displays the overall genetic pattern of each thymoma patient and the central orange horizontal line represents the common genetic alterations of 8,411 genes in all patients. (A) Overall microarray-CGH profile of 39 thymoma patients showing the losses on chromosomes 1, 2, 3, 4, 5, 6, 8, 12, 13 and 18. (B) The common genetic alteration patterns in type A with losses of chromosomes 2, 4, 6q, and 13. (C) The common genetic alteration patterns in type B1 with losses of chromosomes 1p, 2q, 3q, 4, 5, 6q, 8, 13, and 18 and a gain of chromosome 9q.

For evaluating the pattern of genetic aberration of thymoma, 13,248 unique genes were obtained after preprocessing, of which 8,411 were mapped by SOURCE. Commonly amplified or deleted regions were identified by averaging the log_2 _ratio values of each gene from 39 thymoma patients, plotted on the location of the chromosomes using the Chromosome Analyzer and Map Viewer using S-plus (CAMVS) (Figure 1A). When we evaluated overall chromosome patterns based on the cut-off value of ± 0.3, deletions in chromosomes 1, 2, 3, 4, 5, 6, 8, 12, 13 and 18 were identified.

**Figure 2:**
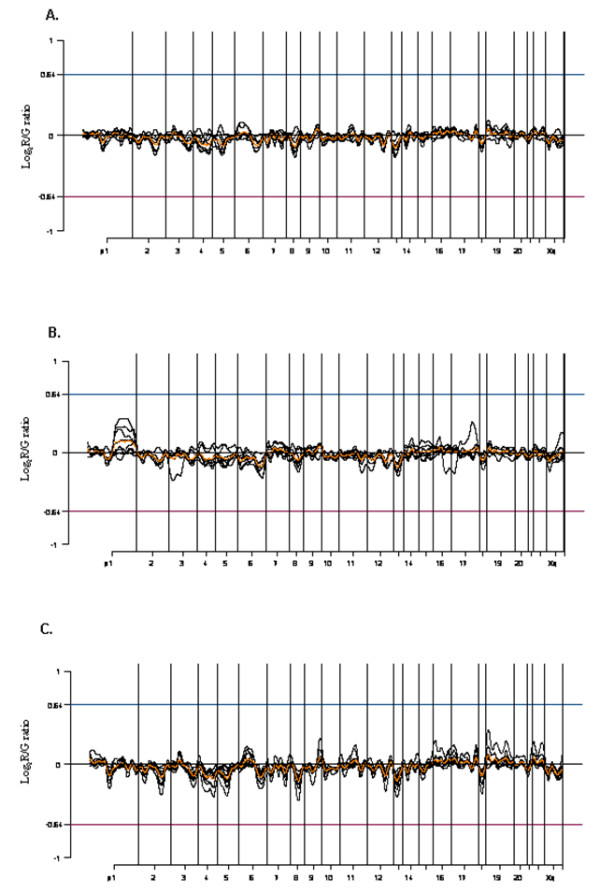
(A) The common genetic alteration patterns in type B2 with losses of chromosomes 1p, 2q, 3q, 4, 5, 6q, 8, 13, and 18. (B) The common genetic alteration patterns in type B3 with losses of chromosomes 2q, 4, 5, 6, 8, 12q, 13, 18 and a gain of chromosome 1q. (C) The common genetic alteration patterns in type AB with losses of chromosomes 2, 4, 5, 6q, 8, 13 and 18.

Subtype specific analysis demonstrated losses of chromosomes 2, 4, 6q, and 13, identified in type A (Figure 1B), and losses of chromosomes 1p, 2q, 3q, 4, 5, 6q, 8, 13, and 18 and a gain of chromosome 9q were identified in type B1 (Figure 1C). Type B2 demonstrated losses of chromosomes 1p, 2q, 3q, 4, 5, 6q, 8, 13, and 18 (Figure 2A), and type B3 revealed chromosome 2q, 4, 5, 6, 8, 12q, 13, and 18 losses, and chromosome 1q gain (Figure 2B). Type A therefore demonstrated the least degree of chromosomal abnormality while subtypes of type B thymoma showed overlapping pattern losses in many chromosomes. Chromosome 9q gain was identified only in type B1 thymoma, while chromosome 1q gain was present only in type B3 thymoma. Meanwhile, type AB showed the losses of chromosomes 2, 4, 5, 6q, 8, 13 and 18 (Figure 2C). In addition, losses of chromosomes 2, 4, 6, and 13 were observed in all subtypes.

### Overall genetic pattern analysis

**Figure 3:**
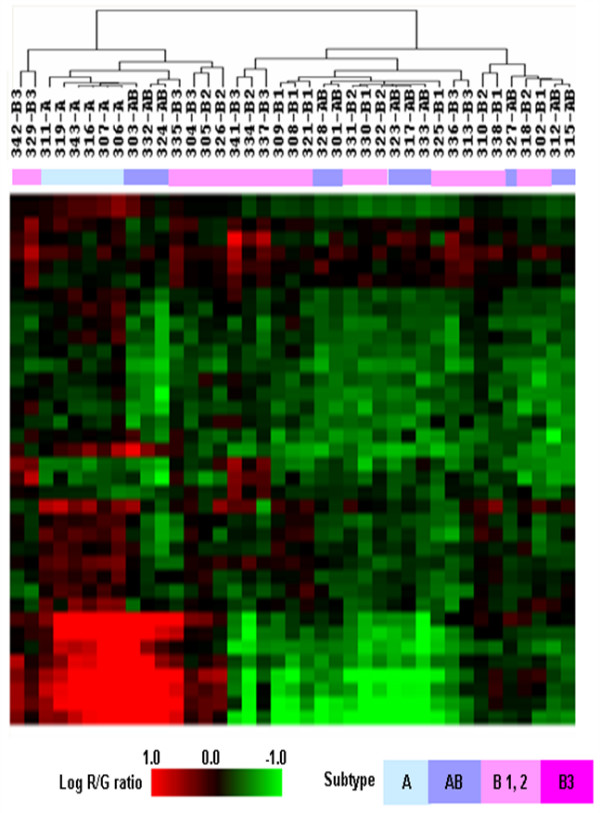
Genetic pattern profiles of thymoma subgroups by the hierarchical clustering of 36 selected genes. Color scale bar showing the level of log R/G ratio. The thymoma subgroup of each patient is displayed in a different color as below.

To investigate the genetic differences of five subtypes of thymoma, we selected 36 distinctive genes at a false discovery rate (FDR) of 2.5% by multi-class SAM (23) (Additional file 1). Supervised hierarchical clustering of 39 cases of thymoma microarray-CGH data with the selected 36 genes demonstrated that types A and B were separately clustered (Figure 3). However, types B1, B2 and B3 branches were intermingled with each other. In addition, eight samples of type AB were dispersed in various B branches, and three samples of type AB were included in type A. These results suggest that type AB has combined characteristics of types A and B, genetically (Figure 3). Hence, type AB was excluded from further analyses to diminish error rate in understanding the genetic characteristics of thymoma subtypes. Among the 36 selected genes, 16 are ESTs with unknown functions and six are located in the 1q region. Cervical cancer oncogene-4 (HCC-4, 2q24.2) was amplified in all subtypes except for type B1. T-cell receptor gamma locus (TARP, 7p15-p14) was deleted in all subtypes except for type A (AB: 63%, B1: 33%, B2: 50%, B3: 12.5%).

### Comparison of genetic aberration patterns between subtypes of thymoma

#### A) Comparison between type A and type B

**Figure 4:**
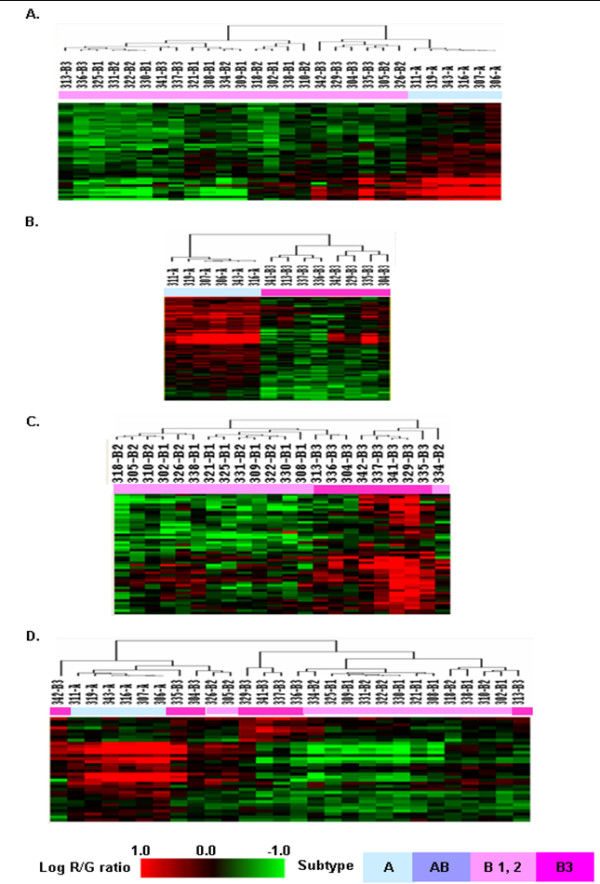
Comparison of genetic aberration patterns between subtypes of thymoma. (A) Hierarchical clustering with 50 selected genes showing different genetic patterns between types A and B (1, 2, 3). (B) Hierarchical clustering of 70 genes showing different genetic patterns in types A and B3. (C) Hierarchical clustering of 48 genes showing the different genetic patterns between types B (1, 2) and B3. (D) Hierarchical clustering with 33 genes showing three molecular characteristic subgroups of thymoma as types A, B(1,2) and B3.

SAM was performed with types A and B in order to identify genetic alterations distinguishing tumors composed of medullary and cortical epithelium. We selected 50 genes at a FDR of 1.7%, and the hierarchical clustering showed the clear separation of type A and type B (Figure 4-A, Additional file 2). Small branch pattern analyses showed more similarity between type B1 and type B2 than between type B2 and type B3, and some similarity between type A and type B3. Among the 50 genes selected, chromosomes 1 and 5 included 10% each (5/50), and chromosomes 6, 11, 13 and 17 each included 6% (3/50). We observed that these genes were closely located within each chromosome, suggesting the possible fragmental losses of thymoma chromosomes. For example, three genes were located nearby on 1p31, and four genes were located nearby on the 5q arm. Among the genes on chromosome 1, FLJ20489 (10/22), 15 KDa selenoprotein (SEP15, 1p 31, 6/22), leptin receptor gene related protein (LEPR, 1p 31, 7/22), and phosphatidylinositol glycan, class K (PIGK, 1p 31, 11/22), were frequently deleted in cortical subtypes.

#### B) Comparison between type A and type B3

SAM was performed with types A and B3 in order to identify genetic alterations responsible for the markedly different biologic behaviors of types A and B3. Seventy genes with different copy numbers were selected with a FDR of 1.2%, and the cluster analysis using these 70 genes showed clear separation between type A and type B3 (Figure 4-B, Table 1). Gains of these genes were prevalent in type A while losses were prevalent in type B3. Twenty-five of the 70 genes were ESTs, and 12 genes were located on chromosome 6, most of which were concentrated on the 6p21.3-6p25 regions. In type B3, frequent deletions were observed in chromosome 6 open reading frame 10 (C6orf10, 6p21.3), butyrophilin subfamily 3, member A2 (BTN3A2, 6p22.1), and thiopurine s-methyltransferase (TPMT, 6p22.3), with a frequency of 75% (6/8), 87.5% (7/8), 75% (6/8), respectively. All the selected genes on chromosome 5 were on the 5q arm, and we observed that cervical cancer oncogene 4 (HCC-4, 2q24.2) was more amplified in all type A cases than type B3 cases.

**Table 1:** List of distinctive 70 genes between types A and B 3

ID	Symbol	Cytoband	Name	type A		type B3	
				gain	loss	gain	loss

AA186327	SNX14	6q14-q15	sorting nexin 14				(5/8)
AA458945	RAB30	11q12-q14	RAB30, member RAS oncogene family	(5/6)			(4/8)
AI201652	EST			(4/6)		(1/8)	(3/8)
AI337344	HSRG1	16q23.1	HSV-1 stimulation-related gene 1	(6/6)		(3/8)	(1/8)
AA991868	EST			(6/6)		(3/8)	(2/8)
AA465687	HCC-4	2q24.2	cervical cancer oncogene 4	(6/6)		(2/8)	(4/8)
AI493835	ESTs			(6/6)		(2/8)	(1/8)
AW087220	ESTs			(4/6)			
AA478436	SMARCD2	17q23-q24	SWI/SNF related, matrix associated, actin dependent regulator of chromatin, subfamily d, member 2	(6/6)			(2/8)
AI015542	TG737	13q12.1	Probe hTg737	(5/6)		(2/8)	
AI733279	ESTs			(6/6)		(2/8)	(4/8)
AA973681	ESTs			(3/6)			
N93021	TBCA	5q14.1					(5/8)
AA917489	ESTs			(6/6)			
AA775364	RPL30	8q22	ribosomal protein L30	(1/6)			
AA987488	ESTs			(2/6)			(2/8)
AA478585	BTN3A3	6p22.1	butyrophilin, subfamily 3, member A3	(1/6)			(2/8)
AI291184	OCLN	5q13.1	occludin	(5/6)			(2/8)
AI348521	HBS1L	6q23-q24	HBS1-like				(6/8)
AA287917	TNPO1	5q13.2	karyopherin (importin) beta 2	(2/6)			(2/8)
AA885609	MAP1A	15q13-qter	microtubule-associated protein 1A				(8/8)
AI375415	ESTs						(2/8)
AA489670	ESTs						(3/8)
AI972677	MSMB	10q11.2	microseminoprotein, beta-	(1/6)			(1/6)
N71628	SPIB	19q13.3-q13.4	Spi-B transcription factor (Spi-1/PU.1 related)	(3/5)			(1/6)
AI383789	ESTs						
AI261862	ESTs			(2/6)			(3/8)
AI985214	TFPI	2q31-q32.1	tissue factor pathway	(2/6)			
H17513	HSPA1L	6p21.3	heat shock 70kDa protein 1-like				(2/8)
T61428	NEDD9	6p25-p24	neural precursor cell expressed, developmentally down-regulated 9	(2/6)			(2/8)
N90109	NCL	2q12-qter	nucleolin	(3/6)			(1/8)
AI972568	RPL36A	Xq22.1	ribosomal protein L36a	(3/6)			
AA442092	CTNNB1	3p21	catenin, beta 1, 88 kDa				(1/8)
AA902196	ESTs			(2/6)			(1/8)
AW009090	NDUFB3	2q31.3	NADH dehydrogenase 1 beta subcomplex, 3, 12 kDa				(1/8)
AA975250	MAT2B	5q34-q35.1	methionine adenosyltransferase II, beta				(3/8)
AI281237	C13orf12	13q12.3	chromosome 13 open reading frame 12				(3/8)
H51066	OBRGRP	1p31.3	leptin receptor gene-related protein				(2/8)
AA465386	DDX21		DEAD (Asp-Glu-Ala-Asp) box polypeptide 21				(3/8)
AI951501	RPL12	9q34	ribosomal protein L12	(6/6)		(1/8)	
AW075424	SFRS3	6p21	splicing factor, arginine/serine-rich 3	(6/6)			(1/8)
AI086414	EST						(2/8)
AI279626	EST						(2/8)
AA677257	TPMT	6p22.3	thiopurine S-methyltransferase				(6/8)
AA887201	C13orf1	13q14	chromosome 13 open reading frame 1				(1/8)
AI284218	ESTs			(2/6)			(1/8)
AA991871	FOXA1	14q12-q13	forkhead box A1	(3/6)			
AA864434	ESTs			(4/6)		(1/8)	(1/8)
AI318162	FLJ14936	1p33-p32.1	hypothetical protein FLJ14936				(1/8)
AA504351	ZNF146		zinc finger protein 146		(1/6)		(6/8)
AI302566	ESTs			(3/6)			(1/8)
AI311303	ESTs						(3/8)
AW078798	UBB	17p12-p11.2	ubiquitin B	(6/6)		(2/6)	
AA927666	ARCH	1p35.1	archease	(3/6)			(1/8)
AA426027	SNX3	6q21	sorting nexin 3	(3/6)			(6/8)
AI262055	ESTs						(1/8)
AA936514	C6orf10	6p21.3	chromosome 6 open reading frame 10		(1/6)		(6/8)
AI364310	ESTs						(5/6)
AA504141	UBE2J1	6q15	ubiquitin-conjugating enzyme E2, J1 (UBC6 homolog, yeast)				(1/8)
AA130187	WT1	11p13	Wilms tumor 1	(1/6)			
N56693	COX7B	Xq21.1	cytochrome c oxidase subunit VIIb	(5/6)		(1/8)	
AA401441	BF	6p21.3	B-factor, properdin				(1/8)
AA917821	MRPS30	5q11	mitochondrial ribosomal protein S30	(1/6)			
AI431827	ESTs						(4/8)
AA935697	ESTs						(3/8)
AI248605	BTN3A2	6p22.1	butyrophilin, subfamily 3, member A2				(7/8)
AI206970	ESTs						
AW058415	HBXIP	1p13.3	hepatitis B virus × interacting protein	(2/6)			
N69107	YWHAH	22q12.3	tyrosine 3-monooxygenase/tryptophan 5-monooxygenase activation protein, eta polypeptide				(1/6)
AA863025	ESTs			(2/6)			

The ID indicate GeneBank ID and the symbol and the cytoband information are from their SOURCE . The incidence is the number oh cases having log2 ratio in the range of our criteria ± 0.3. ESTs are expressed sequenced tags, clones of unknown functions. Genes are listed according to order FDR values.

#### C) Comparison of cortical subtypes

Based on the previous results demonstrating that among cortical subtypes, the pattern of small branches showed more similarity between types B1 and B2 than types B2 and B3, we divided cortical subtypes into types B1+B2 and B3 for evaluating genetic alterations. We selected 48 significant genes with a FDR of 11% by SAM (Figure 4-C, Additional file 3). As the FDR value of differential gene selection is relatively high, type B1+B2 and type B3 were not clustered clearly, but they showed a tendency of separation. Twenty-nine of 48 selected genes were on chromosome 1, and nine of them were ESTs. Among the 29 genes on chromosome 1, 26 (86%, 26/29) were amplified in type B3 type, while only four (13%, 4/29) were amplified in type B1+2. The selected genes were concentrated in the 1q32 and 1q42-q43 regions.

#### D) Comparison between type A, type B 1+2, and type B3

SAM was carried out with three subgroups of types A, B1+B2, B3, and 33 significant genes were selected at 2.6% FDR, most of which are located on chromosomes 1 and 6 (Table 2). When a clustering analysis was carried out with these 33 genes (Figure 4-D), type A was clearly separated from types B1+2 and B3.

**Table 2:** List of distinctive 33 genes among types A, B (1, 2) and B3

ID	Symbol	Cytoband	Name	type A		type B (1,2)		type B3	
				
				gain	loss	gain	loss	gain	loss
AA186327	SNX14	6q14-q15	sorting nexin 14				(1/14)		(5/8)
AA287917	TNPO1	5q13.2	karyopherin (importin) beta 2	(4/6)			(1/14)		(2/8)
AA442092	CTNNB1	3p21	catenin, beta 1				(1/14)		(1/8)
AA455917	SEC22L1	1q21.2-q21.3	SEC22 vesicle trafficking protein-like 1		(2/6)		(8/14)	(2/8)	(1/8)
AA458945	RAB30	11q12-q14	RAB30, member RAS oncogene family	(5/6)			(10/14)		(4/8)
AA465386	DDX21		DEAD (Asp-Glu-Ala-Asp) box polypeptide 21				(4/14)		(3/8)
AA465687	RBMS1	2q23.2	cervical cancer oncogene 4	(6/6)		(2/14)	(7/14)	(2/8)	(4/8)
AA478436	SMARCD2	17q23-q24	SWI/SNF related, matrix associated, actin dependent regulator of chromatin, subfamily d, member 2	(6/6)		(3/14)	(6/14)	(1/8)	(2/8)
AA478585	BTN3A3	6p21.3	butyrophilin, subfamily 3, member A3	(1/6)					(1/8)
AA774608	PPP1R12B	1q32.1	protein phosphatase 1, regulatory (inhibitor) subunit 12B					(1/8)	
AA931882	ESTs					(3/14)		(5/8)	
AA973568	ESTs			(6/6)		(1/14)	(9/14)	(2/8)	(4/8)
AA973681	ESTs			(5/6)			(3/14)		(1/8)
AA991868	ESTs			(6/6)		(2/14)	(7/14)	(3/8)	(1/8)
AA991871	FOXA1	14q12-q13	forkhead box A1	(3/6)		(1/14)		(3/8)	
AI001856	ESTs		BRG1-binding protein ELD/OSA1	(2/6)			(6/14)		(2/8)
AI015542	TTC10	13q12.1	Probe hTg737	(5/6)			(10/14)	(2/8)	(1/8)
AI201652	ESTs			(5/6)			(7/14)	(1/8)	(3/8)
AI261862	ESTs			(2/6)			(12/14)		(3/8)
AI289196	MAN1A2	1p13	mannosidase, alpha, class 1A, member 2	(3/6)					
AI291184	OCLN	5q13.1	occludin	(5/6)		(2/14)	(1/14)		(1/8)
AI300984	ESTs			(1/6)			(2/14)		
AI311303	ESTs						(9/14)		(3/8)
AI335359	C1orf21	1q25	chromosome 1 open reading frame 21					(3/8)	
AI337344	HSRG1	16q23.1	HSV-1 stimulation-related gene 1	(6/6)		(1/14)	(8/14)	(3/8)	(2/8)
AI348521	HBS1L	6q23-q24	HBS1-like				(8/14)		(6/8)
AI392929	PLU-1	1q32.1	putative DNA/chromatin binding motif					(1/8)	
AI401608	ESTs							(2/8)	
AI492063	ESTs				(2/6)	(1/14)	(3/14)	(5/8)	
AI493835	ESTs			(6/6)		(1/14)	(4/14)	(2/8)	(1/8)
AI655101	XPR1	1q25.1	xenotropic and polytropic retrovirus receptor			(1/14)	(1/14)	(4/8)	
AI972955	TARP	7p15-p14	T cell receptor gamma locus	(1/6)			(7/14)		
H99842	EIF5A	17p13-p12	eukaryotic translation initiation factor 5A	(2/6)					

The ID indicate GeneBank ID and the symbol and the cytoband information are from their SOURCE . The incidence is the number oh cases having log_2_ratio in the range of our criteria ± 0.3. ESTs are expressed sequenced tags, clones of unknown functions. Genes are listed according to order FDR values.

Twelve of the selected 33 genes were ESTs, with six genes on chromosome 1. Four genes located on 1q, chromosome 1 open reading frame 21 (C1orf21), putative DNA/chromatin binding motif (PLU-1), protein phophatase1 regulator subunit 12B (PPP1R12B), and SEC22 vesicle trafficking protein-like 1 (SEC22L1) were amplified only in type B3.

#### E) Prediction analysis of thymoma subtypes

**Figure 5:**
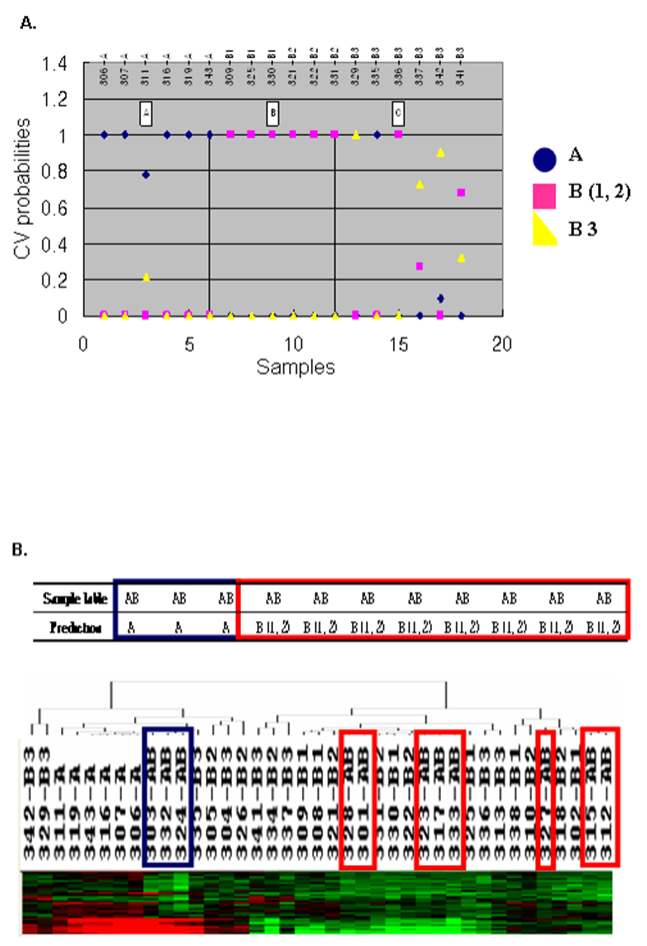
Prediction analysis of thymoma subgroups. (A) Cross-validated probabilities of the selected 136–44 genes at threshold 2.78 in the training set using 18 cases of thymoma subgroups, six cases each of A, B (1, 2), B3, showing 90% accuracy. (B) Prediction analysis of 11 ambiguous type AB samples as a training set showed the similar result as Figure 2, designating three as type A and eight as type B.

Based on the previous results, thymoma could be divided into three genetically distinct subgroups (types A, B1+B2, and B3) and genetically heterogeneous type AB. Prediction Analysis of Microarray (PAM) (24) as in the method was carried out to predict to which subgroup type AB belongs. To equilibrate each group, the training set evenly included six cases from types A, B1+2, and B3. Eleven cases of type AB were used as a test set in the prediction analysis. The cross-validation of the selected 44 subgroup classifier genes showed 100% accuracy with types A and B1+B2 and 50% accuracy with type B3 in the training set (Figure 5-A). The prediction analysis demonstrated that among the 11 cases of type AB, three cases were classified into type A, and the remaining eight cases into type B (1, 2) (Figure 5-B), which is coherent with the dendrogram in Figure 3.

## Discussion

A number of study results on systemic genetic analyses of thymoma have been reported in the past few years after a few case reports on the chromosomal abnormalities of thymoma [19-22], [25-27]. Zettl et al. demonstrated for the first time recurrent chromosomal imbalances in type B3 thymoma with CGH and FISH methods [19]. They identified 16 cases of type B3 that showed chromosomal gains (1q, Xq, and 8p12) and losses (6 and 13q), while 12 cases of type A did not reveal any chromosomal abnormalities, with the exception of one case with a partial loss of 6p. Subsequently, the same group, using CGH and microsatellite analyses, inferred two pathways of thymoma tumorigenesis by demonstrating that type A (3/8 cases) presented with consistent LOH in the region 6p23.3-25.5 only, while type B3 revealed various aberrations such as APC on chromosome 5q21 (3/14 cases), RB on 13q14 (5/14 cases), and p53 gene on 17p13.1 (4/14 cases) loci, as well as LOH in the region 6p23.3-25.5 (5/14 cases) [20]. They expanded their samples to include types AB and B2 in addition to types A and B3 by using laser-assisted microdissection or short-term thymic epithelial tumor cell cultures and found that 1) the various WHO-defined subtypes of thymoma exhibited different profiles of genetic alterations, 2) only type A was genetically homogeneous with abnormalities mainly involving chromosome 6 while other subtypes showed genetic heterogeneity, and 3) some cases of type B2 were genetically closely related to type B3, and might arise from this type by gain of genetic aberrations [21]. In addition, Penzel et al [22] reported a main cluster characterized by a gain of 1q and losses of 6q and 16q occurring only in type B3 (3/4 cases). Three of eight cases of type A thymoma demonstrated various types of chromosomal imbalances in contrast to the study results reported by Zetti et al [19]. Namely, the results of the CGH studies on thymoma by two groups demonstrated some discrepancies [19-22].

We used cDNA microarray based-CGH to investigate the genome-wide genetic aberrations of thymoma. In contrast to CGH and LOH analyses, cDNA microarray based-CGH could provide us a high-throughput evaluation of the whole genome and also with significant genetic information at a single gene level. The other advantages of using microarray-CGH are the utility of paraffin embedded tissue DNA, the requirement of a small amount of genomic DNA and the possible direct comparison with RNA expression. With this technique there was no need to amplify the DNA in order to obtain the genetic information of more than 10,000 genes, which sometimes may blur the results. Compared to RNA expression analysis, for which we need the fresh, well-stored tissue samples (the major limitation of tissue procurement), using the paraffin embedded tissues allowed us to do the retrospective studies using a large number of samples.

The main purpose of our study was to identify differential genetic patterns to explain distinct morphologic findings and different biologic behaviors, according to subtypes of thymoma. For this, we included all WHO-defined subtypes of thymoma for genetic analysis, as in the previous reports [25-29]. The previous studies using CGH and microsatellite analyses excluded subtypes with large numbers of lymphocytes (type AB, B1, and B2) from the samples because CGH analyses generally require a tumor cell content of more than 50% [19,20]. As we consider the clinicopathological behavior of thymoma comes from the complex biology with thymic spindle epithelial cells and surrounding microenvironment including lymphocytes. Hence, to understand the phenotypic biology of thymoma, whole tissues were used for genetic evaluation in this study. The results from the current data might support in-depth understanding of tumor biology with more clinically relevant information, resulting in potential clinically useful biomarker candidates for subtype classification. However, the careful explanation is needed for understanding pathogenesis of thymoma concerning the influence from the lymphocytes.

The CAMVS, which was developed in our institute for the effective analysis and visualization of microarray-CGH results, was used to identify the significant genetic aberrations of thymoma. Chromosomal deletions in chromosomes 1, 2, 3, 4, 5, 6, 8, 12, 13 and 18 were observed by the comparison of the mean log_2 _ratio after microarray-CGH of a total of 39 cases, suggesting the characteristic genetic aberrations of thymoma. The chromosome 6 loci, known to possess many tumor suppressor genes, well-established deletion sites in thymoma such as 6q21, 6q23, and 6q25-27, and the genes selected in our study, were also frequently located on 6q, confirming the previous data [29].

Subtype specific analyses demonstrated that losses of chromosomes 2, 4, 6, and 13 were identified in all subtypes of thymoma. Type A thymoma had the least number of chromosomal abnormalities while type B had many more chromosomal abnormalities in accordance with the previously reported data [19-22]. Thymoma type B revealed increased genetic aberrations suggesting a rupture in chromosomal stability, and these findings correlate well with the indolent biological behavior of type A and the aggressive behavior of type B. Although a loss of chromosome 6q had been reported as the only chromosomal abnormality in a rare case of type A thymoma, a recent report by Penzel et al [22] identified various types of chromosome gains such as 1q, 9q, 16, 17, 20, and 22 and losses such as 2q, 4q, 5q, 6q, 9p, and 13q in three out of eight cases of type A analyzed. We also identified losses of chromosomes 2, 4, 6q, and 13 in type A. So the results of our study support the genetic heterogeneity in a significant proportion of type A, although genetic aberrations were usually less prominent, as compared with type B.

Furthermore, type B demonstrated heterogeneous and overlapping chromosomal aberrations in our study. Losses of chromosomes 1p, 2q, 3q, 4, 5, 6q, 8, 13, and 18 and a gain of chromosome 9q were identified in type B1. Type B2 demonstrated losses of chromosomes 1p, 2q, 3q, 4, 5, 6q, 8, 13, and 18 and type B3 revealed chromosome 2q, 4, 5, 6, 8, 12q, 13, 18 losses and chromosome 1q gain. We could confirm the type B3 specific 1q gain and losses of 6, 13, and 16 in all three cortical subtypes, as in previous reports [19-22]. However, losses of chromosomes 2q, 4, 5, 8, 13 and 18 were also identified in all three cortical subtypes. So our results also confirm the presence of various other overlapping chromosomal abnormalities in three cortical subtypes in addition to well established B3 specific 1q gain and chromosome 6, 13, and 16 losses. Furthermore, we identified chromosomal abnormalities of type B1 for the first time. Type B1 shared a similar pattern of chromosome losses with B2 but showed a 9q gain, which was identified only in type B1. These diverse genetic variations in our data support the underlying genetic influence on the biology of thymoma subtypes, resulting in various clinical behaviors.

After the systematic insight into genetic aberrations of thymoma was achieved according to molecular characteristics, the types were dissected into genetically related subgroups for further detailed analyses. First, we identified that type AB is genetically heterogeneous, so we excluded type AB for further analyses. Then, we identified that type A was distinct from type B, based on the molecular characteristics.

The cortical subtypes did not demonstrate clear separation by hierarchical clustering analysis. Among cortical subtypes, type B1 morphologically maintains distinct cortico-medullary differentiation with indistinct tumor epithelial cells and usually shows indolent biologic behavior, as compared with types B2 and B3. Type B2, which definitely shows more aggressive biologic behavior than type B1, has more prominent tumor epithelial cells than type B1 and gray zones are sometimes present between type B2 and B3 histologically. Furthermore, tumors having both type B2 and B3 areas are commonly present. So we expected genetic similarities between type B2 and type B3. However the pattern of small branches showed more similarities between types B1 and B2 than between types B2 and B3.

Based on the hierarchical clustering analysis, we could assume that thymoma could be divided into four genetically different subgroup of types A, AB, B1+2, and B3. We tried to assign 11 genetically heterogeneous cases of type AB into types A and B using the prediction analysis. The prediction analysis results were coherent with the clustering analysis results, showing that three clustered in type A were predicted to be type A, and eight clustered in type B were designated as type B. Type AB was reported to be genetically more heterogeneous than type A and some chromosomal aberrations characteristic of type B were reported to be present in type AB [21,22]. Type AB is defined as an organotypic thymoma, showing both features of medullary and cortical thymoma. In fact, there is a wide morphologic spectrum in type AB. Some show a distinct nesting of spindled epithelium as in the Regard type of nasopharyngeal carcinoma, while in others, epithelial cells are sprinkled individually as in the Schmincke type of nasopharyngeal carcinoma.

The WHO classification of thymoma is reported to be associated with the invasiveness and recurrence of thymoma. Among the various gene sets, the 70 genes distinguishing types A and B3 might be related to the malignancy of thymoma, because B3 is the typical malignant tumor with a metastatic property among 5 subtypes. Among the 70 genes, the significant number of genes were related to cell structure and adhesion. NEDD9 (T61428, neural precursor cell expressed developmentally down regulating 9) and CTNNB (AA442092, cadherin-associated protein beta 1), which are known to be the adhesion-related genes, were deleted in type B3, suggesting increased cell motility for metastasis. As Penzel et al. reported the genetic amplification in chromosome 1 in malignant type B2 and B3, frequently amplified genes chromosome 1 were observed in B3 [22].

There have been no standard methods to detect the significant genes among the distinct groups in microarray-CGH. Several reports mentioned the correlation of the CGH and expression profiling suggesting the possibility of using similar approach for both in selecting the significant genes [33,34]. Among the several analytic methods for gene expression profiling, SAM method has been used as one of the standard methods based on the t-test. Therefore, SAM method is applied in this investigation. Recently, a few reports suggest new methods to detect numbers of significant genes between distinct groups in microarray-CGH [35,36]. As there is no valid method to analyze differentially gained or lost genes in microarrya-CGH, more appropriate method with biological validation should be evaluated.

Only one concern of this study is the limited numbers of the samples in each type. Especially with the microarray-CGH, which could evaluate thousands of genes simultaneously, the sample size is the significant matter. To overcome the over-fitting problem, one effort is to divide the samples into the training and the independent test set. However, the sample size is not large enough to select the genes based on this approach, requiring us to apply the cross-validation method in the same training set. As all these efforts to define the subgroups based on the clinicopathologic or molecular level result from the lack of good prognostic markers and treatment strategies of thymoma, the capacity of prediction of current proposed genes sets needs to be validated in more samples prospectively in accordance with the clinical parameters.

## Conclusion

In this study, we evaluated the genetic characteristics of the WHO-defined five subtypes of thymoma using microarray-CGH. We observed that thymoma could be divided into four genetically distinct groups of A, AB, B1+2, and B3. Type AB was determined to be genetically heterogeneous in morphology. We identified sets of genes which characterize the molecular subgroups for the basis of understanding thymoma biology and the candidate biomarkers of each group. In conclusion, this study provides significant information on the genetic background of thymoma for classification purposes.

## Methods

### Patients

Thirty-nine cases of thymoma tissue samples (A : 6, AB : 11, B1 : 7, B2 : 7, B3 : 8) were obtained as paraffin embedded tissue blocks from the files of the Department of Pathology at the Severance Hospital, Yonsei University College of Medicine. The median age of patients at diagnosis was 50 years (range 26–71 years), and the male versus female ratio was 23:16.

### DNA preparation

Ten serial 10 μm thick tissue sections were cut from representative paraffin blocks for genomic DNA extraction. The pathologist (Professor Yang WI) confirmed the diagnoses and subtypes according to the WHO classification (9), and localized tumor areas in the corresponding hematoxylin-eosin (H&E) stained tissue section. Fresh normal placental tissues from healthy newborns were snap-frozen for reference samples. Genomic DNA extraction from the slide was performed according to the conventional protocol[30]. Briefly, scraped tissue from the slides was deparaffinized by washing it twice with 1 μl of xylene at 55°C for five minutes. After two washes with 100% ethanol, the samples were dried for two hours at 50°C. The tissues for reference samples were incubated with 400 μl of DNA lysis buffer [10 mM Tris pH 7.6, 10 mM EDTA, 50 mM NaCl, 0.2% SDS, 200 μg/ml Proteinase K] at 42°C for 12 to -24 hours. The incubated products were treated with the same amount of phenol/chloroform/isoamylalcohol (Gibco-BRL, Gaithersburg, MD, USA) to isolate the nucleic acid from the proteins. The DNA was precipitated with 100% ethyl alcohol containing a 1/3 volume of 10 M ammonium acetate and 2 μl of glycogen. After being rinsed with 70% ethyl alcohol, the DNA was dried at room temperature and then dissolved in ultra-pure water. The quantity and quality of the DNA were evaluated using the Gene Spec III (Hitachi, Japan) and the Gel Documentation-Photo System (Vilber Lourmat, France).

### cDNA microarray based – CGH (Microarray-CGH)

In this study, we used 17K cDNA microarrays (CMRC-GenomicTree, Daejeon, Korea) that included 15,720 unique genes. Of these genes, 11,552 were mapped by SOURCE, a web based database provided by the Genetics Department of Stanford University [37]. Microarray-CGH experiments were performed with the indirect design to determine the genome-wide genetic aberrations in thymoma subtypes using sex-matched placental tissue as a reference. Labeling of DNA was performed following the institutional protocol as described previously [30,31]. Briefly, four μg of placenta and thymoma tissue DNA were fluorescently labeled with Cy3 or Cy5-dUTP (Dupont NEN Life Sciences, Boston, MA, USA), respectively, using BioPrime DNA Labeling System (Invitrogen, Carlsbad, CA, USA). Labeled products were purified with a PCR Purification Kit (Qiagen, Dusseldorf, Germany) and combined with 30 μg of human Cot-1 DNA (Gibco BRL, Gaitherburg, MD, USA), 100 μg of yeast tRNA (Gibco BRL, Gaitherburg, MD, USA) and 20 μg of poly(dA-dT) (Sigma, Saint Louis, MO, USA). Then, the hybridization mixture was concentrated using a Micro-con 30 (Millipore, Bedford, MA, USA) and hybridized to the 17K cDNA microarray at 65°C for 16 to18 hours. After washing, the microarray was scanned using GenePix 4000B (Axon Inc, Foster, CA, USA). The microarray data was obtained by GenePix Pro 4.1 software (Axon Inc, Foster, CA, USA).

### Data Analysis

#### Raw data preprocessing and normalization

Fluorescent spot signals were obtained by subtracting background intensity from the total spot intensity. The genes with missing values for more than one of the experiments were removed for further analysis. The variation from the different labeling efficiencies was corrected using within-slide global normalization, which subtracted the median of log_2 _(R/G) intensity ratio from the log_2 _transformed data.

#### Analysis of Microarray-CGH

The obtained data were analyzed using the Chromosome Analyzer and Map Viewer using S-plus (CAMVS) developed by the Cancer Metastasis Research Center (CMRC), Yonsei University College of Medicine, Seoul, Korea. To evaluate the general genetic pattern of whole chromosome, 0.025 span was introduced to give the weighted mean through the neighboring 250 probes, resulting in the advantage of evaluation of ratios of 250 proves simultaneously. Smoothing line is based on the more weight on the nearer probes. In this study, we used the predetermined cut-off value for the significant copy changes by comparing the level of genetic changes using normal tissue DNAs including placenta, lymphocyte and gastric tissues in the previous study [32]. Based on the assumption that the sex chromosomal single copy difference is the only change between the XX and XY normal gastric tissue DNA, the result showed that the changes in the autosomal chromosomes were minimal with the range of -0.3 < log_2_(R/G) < 0.3 (mean ± 1SD), while the changes in the sex chromosomes were -0.64 < log_2_(R/G)< 0.64. Hence, cut-off values of log_2_(R/G) > ± 0.64 (mean ± 1SD) as amplification or deletion, log_2_(R/G)> ± 0.3 (mean ± 1SD) as gain or loss were determined.

The Significant Analysis of Microarray (SAM) was used to identify the specific genes that showed differences between the groups [23] with more than 15% frequencies in the group. To evaluate the accuracy of selected genes representing the thymoma subtypes, we performed the analysis by using Prediction Analysis of Microarray (PAM) software [24]. The obtained data were clustered using software program Cluster version 2.10 and visualized with Treeview version 1.47 [38].

## Authors' contributions

GYL performed the experiments, data analysis and drafted the manuscript. WIY was participated in case selection and pathologic review. HCJ participated in obtaining the clinical information and data analysis. SCK performed the set-up of analysis program and preliminary analysis MYS performed the set-up of microarray-CGH using gDNA from paraffin-embedded slides. CHP is participated in data analysis. HCC participated in its design and coordination, and data interpretation. SYR conceived of the study, participated in data analysis, interpretation and finalized manuscript. All authors have been read and approved the manuscript.

## Supplementary Material

Additional file 1 Distinctive 36 genes between thymoma 5 subgroups. The ID indicate GeneBank ID and the symbol and the cytoband information are from their SOURCE [37]. The incidence is the number oh cases having log2 ratio in the range of our criteria ± 0.3. ESTs are expressed sequenced tags, clones of unknown functions. Genes are listed according to order FDR values. Click here for file

Additional file 2 List of distinctive 50 genes between types A and B (1, 2, 3). The ID indicate GeneBank ID and the symbol and the cytoband information are from their SOURCE [37]. The incidence is the number oh cases having log2 ratio in the range of our criteria ± 0.3. ESTs are expressed sequenced tags, clones of unknown functions. Genes are listed according to order FDR values. Click here for file

Additional file 3 List of distinctive 48 genes between types B (1, 2) and B3. The ID indicate GeneBank ID and the symbol and the cytoband information are from their SOURCE [37]. The incidence is the number oh cases having log2 ratio in the range of our criteria ± 0.3. ESTs are expressed sequenced tags, clones of unknown functions. Genes are listed according to order FDR values. Click here for file
